# Christian Bauer—physiologist, mentor, and philosopher

**DOI:** 10.1007/s00424-025-03109-0

**Published:** 2025-08-07

**Authors:** Armin Kurtz, Kai-Uwe Eckardt, Max Gassmann, Hugo Marti, Josef Pfeilschifter, Holger Scholz, Roland Wenger

**Affiliations:** 1 Institute of Physiology, University of Regensburg, Regensburg, Germany; 2 Department of Nephrology and Medical Intensive Care, Charité - Universitätsmedizin Berlin, Berlin, Germany; 3 Institute of Veterinary Physiology, University of Zürich, Zürich, Switzerland; 4 Institute of Physiology and Pathophysiology, University of Heidelberg, Heidelberg, Germany; 5 Institute of Pharmacology and Toxicology, University Frankfurt, Frankfurt, Germany; 6 Institute of Translational Physiology, Charité - Universitätsmedizin Berlin, Berlin, Germany; 7 Institute of Physiology, University of Zürich, Zürich, Switzerland

“Of all the wonders that I yet have heard,

It seems to me the strangest that men should fear;

Seeing that death, a necessary end

Will come when it will come”

(William Shakespeare)

**Fig. 1:**
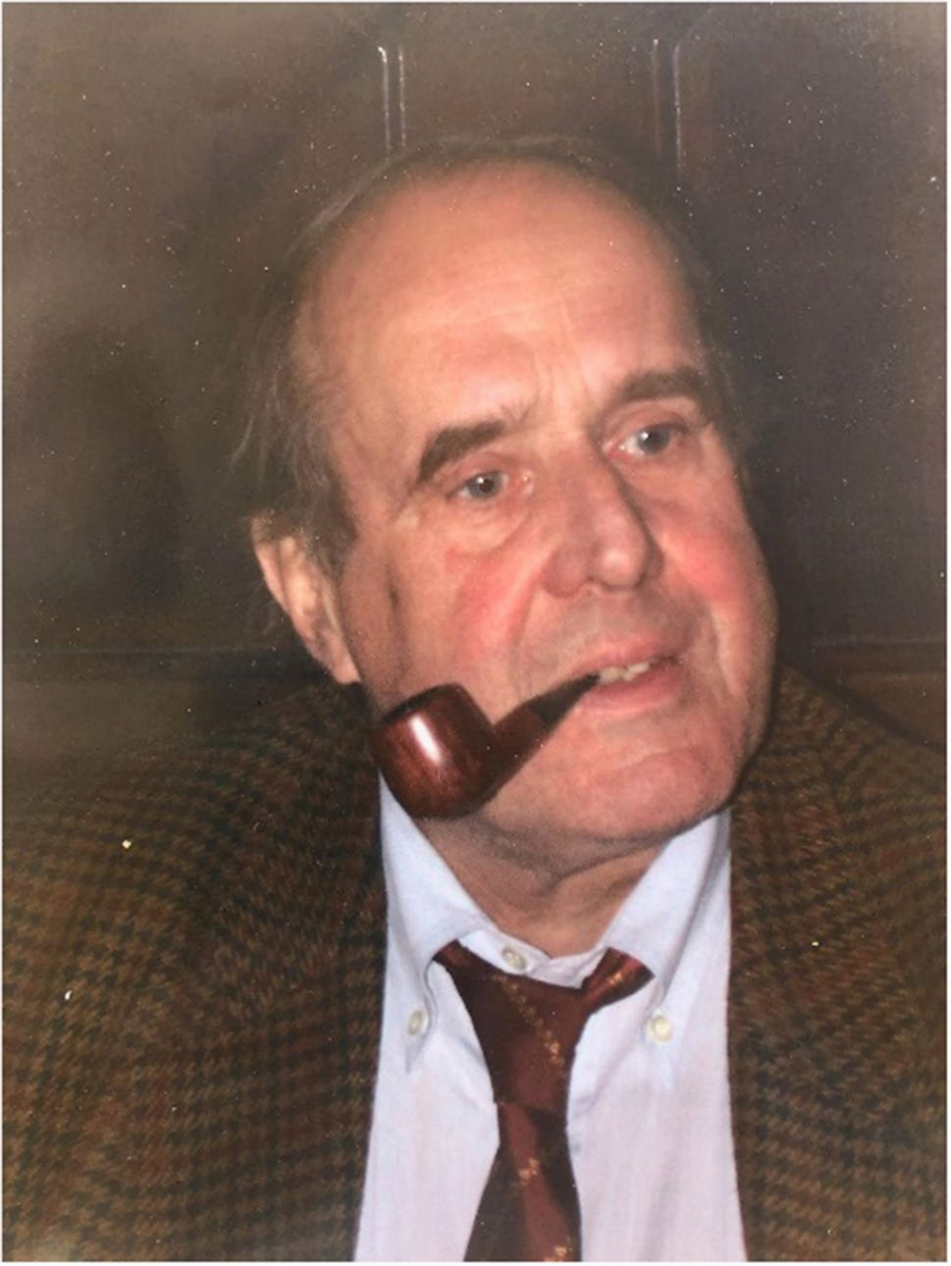
Christian Bauer 20.8.1938–30.6.2025

On June 30, 2025, Emeritus Professor of Physiology Christian Bauer passed away at the age of 86. Christian Bauer was the longtime director of the Physiological Institute at the University of Zurich, Switzerland. With his passing, we bid farewell with gratitude to an ingenious researcher, a caring mentor, and an extraordinary personality (Fig. 1).

Christian Bauer studied human medicine at the Universities of Tübingen and Basel. The subject of his medical doctoral thesis, which he completed in 1965 at the Physiological Institute of the University of Tübingen under the supervision of Prof. Bartels, introduced him to a field of research to which he would remain broadly committed until his retirement in 2003. His thesis entitled “Changes in the electrolyte content in the blood of rabbits during altitude adaptation” sparked his interest in oxygen transport in the blood and its regulation.

In 1967, Christian Bauer followed Prof. Bartels to the Physiological Institute at Hannover Medical School, where he investigated how blood can acutely adapt to situations of reduced ambient oxygen pressure ensuring optimal oxygen supply to tissues, such as in fetuses or at high altitudes. This work led him to the allosteric regulation of hemoglobin’s oxygen affinity by organic phosphates, protons, and carbon dioxide. Organic phosphates such as 2,3-DPG proved to be the strongest influencing factors, significantly reducing the oxygen affinity of hemoglobin and thus facilitating oxygen delivery to the tissue.

In 1971, Christian Bauer qualified as university lecturer in physiology with his habilitation thesis “On the respiratory function of hemoglobin.” After a two-year postdoc in the USA, he was offered a professorship in physiology at the University of Regensburg in 1975. There he studied the function of embryonic hemoglobin and continued his research on the allosteric regulation of hemoglobin. Impressed by the ability of crocodiles, to remain underwater for exceptionally long periods as lung breathers, he explored their hemoglobin function and discovered an exceptionally strong regulation by CO_2_, with organic phosphates playing no role. A spectacular finding that was published in *Nature* in 1977 together with his colleague Wolfgang Jelkmann [1]. In collaboration with the Nobel laureate Max Perutz, he later elucidated the molecular mechanism of action of CO_2_, which led to another Nature publication [2].

After hemoglobin research offered fewer challenges towards the end of the 1970s, Christian Bauer and Wolfgang Jelkmann expanded their research to include not only the oxygen affinity of hemoglobin, but also the hemoglobin concentration and thus the regulation of erythropoiesis as an essential determinant of arterial oxygen transport. Since it was already known at that time that the kidneys produce the erythropoiesis-stimulating hormone erythropoietin under conditions of reduced oxygen supply, Christian Bauer became increasingly interested in the question of where and how oxygen deficiency activates renal erythropoietin production.

In 1984, Christian Bauer was appointed Professor of Physiology and Director of the Physiological Institute at the University of Zurich. Together with Josef Pfeilschifter and his former doctoral student Armin Kurtz, he established a laboratory that focused on the regulation of erythropoietin production in humans and experimental animals. A key technical prerequisite for investigating these questions was the development of a specific radioimmunoassay for erythropoietin, which was successfully pursued by Kai-Uwe Eckardt as a postdoc in the laboratory [3]. A key finding derived from animal experiments was that proximal tubule function plays a key role in the regulation of erythropoietin production during hypoxia [4], even though it is produced in peritubular cells. Holger Scholz, also a postdoc in the laboratory, introduced the model of the isolated perfused kidney to study renal erythropoietin production independently of systemic influences [5].

**Fig. 2:**
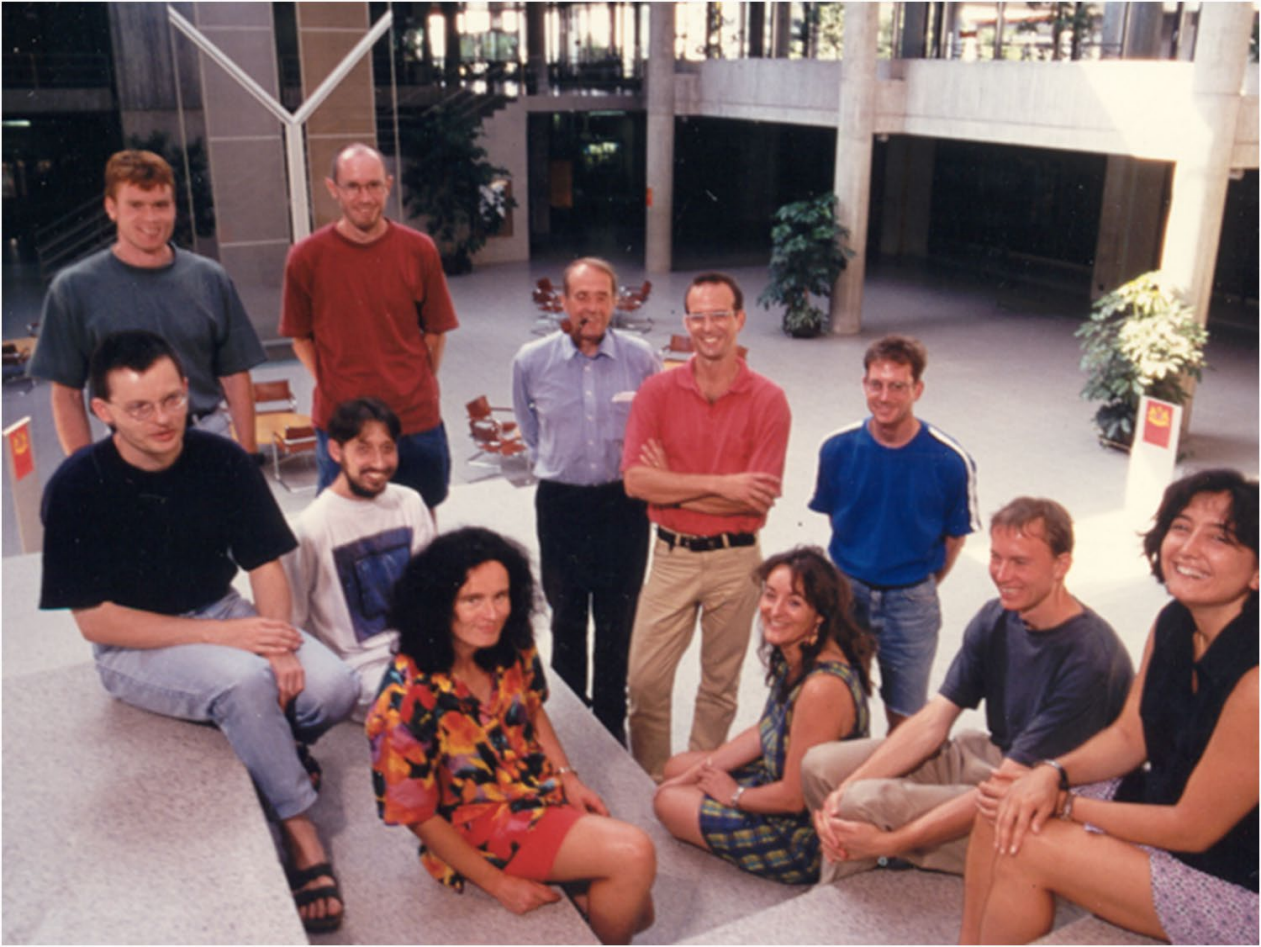
Members of the Bauer lab in Zurich in 1997

After it became clear that erythropoietin formation is transcriptionally regulated, Christian Bauer, together with Max Gassmann, Hugo Marti, and Roland Wenger, turned his attention to the transcription factors that regulate the activity of the erythropoietin gene. His laboratory made numerous contributions to the function and regulation of these “hypoxia-inducible factors.” In this context, they reported the concentration dependency of HIFs on the oxygen tension [6], an induction of acute phase gene expression by hypoxia [7], or the expression of erythropoietin in the brain [2, 8]. Christian Bauer always served as a mentor and granted his colleagues significant scientific freedom. (Fig. 2).

**Fig. 3:**
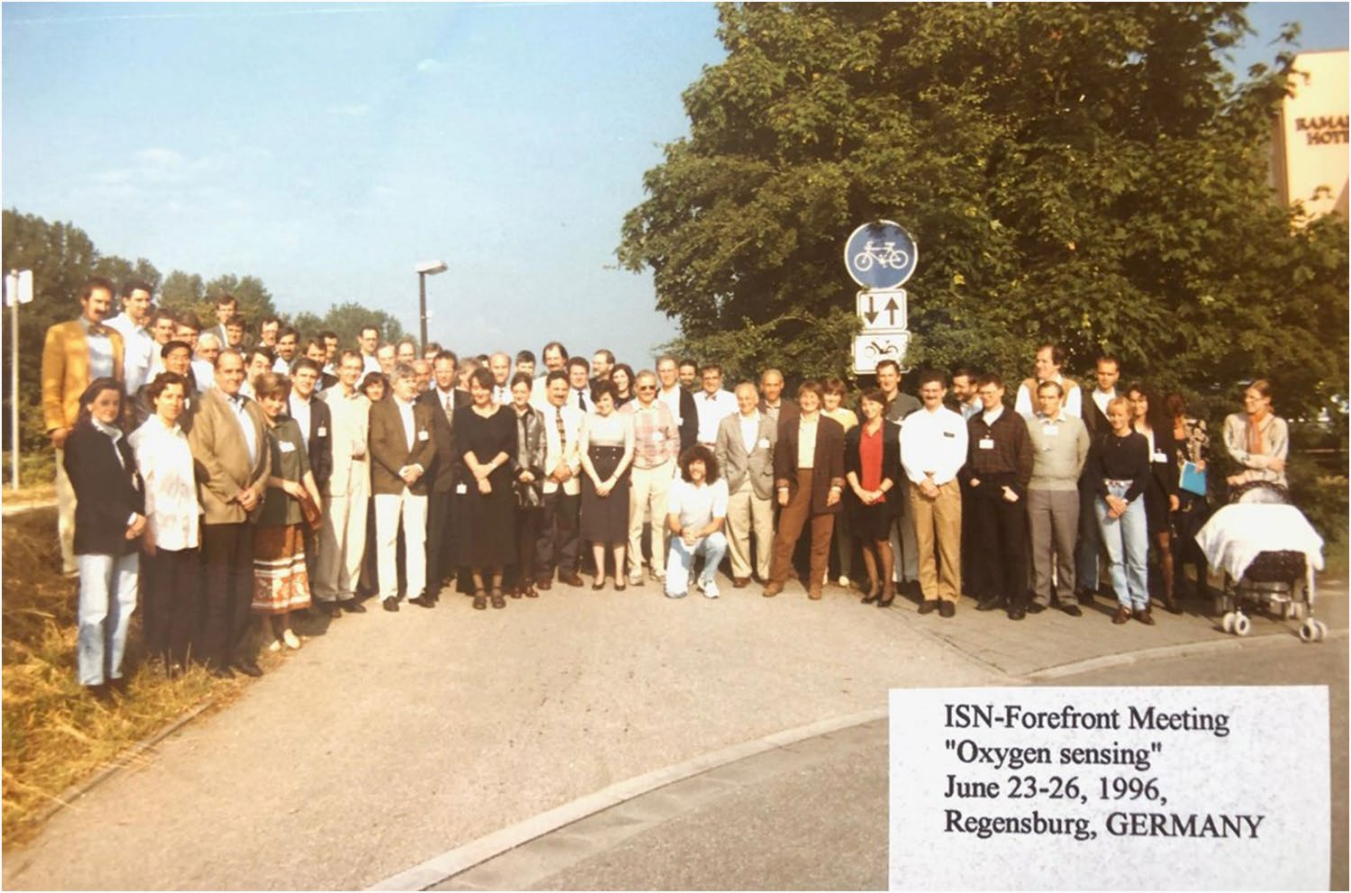
Participants at the ISN-Forefront Meeting on “Oxygen sensing”

Christian Bauer organized several international conferences on the topic of oxygen sensing and erythropoietin. As early as 1992, he hosted a major international symposium on “Erythropoietin: Molecular Physiology and Clinical Applications” in Lucerne, Switzerland, which was also attended by Gregg Semenza and Peter Ratcliffe, who, together with Bill Kaelin, received the 2019 Nobel Prize for their work on oxygen sensing. In 1996, together with Armin Kurtz, he organized a major Forefront Meeting of the International Society of Nephrology in Regensburg on the topic of “Oxygen Sensing on the Cellular and Molecular Level,” which brought together all leading international scientists in the field (Fig. 3).

Christian Bauer also served professional societies. For seven years, he held the position of Secretary General of the Federation of European Physiological Societies (FEPS). In 1996, he hosted the annual congress of the German Physiological Society in Zurich.

Christian Bauer was a generous, widely curious and deeply human mentor who inspired and generously supported his colleagues to pursue their own scientific development. The impressive number of professors who emerged from his research group attests to and rewards this commitment.

Also, beyond science, Christian Bauer was an extraordinary individual. He honored Judaism and spent a sabbatical in Jerusalem to deepen his understanding of Jewish culture. He was profoundly interested in the Old and New Testaments, and he had a passion for mathematics, physics, and astrophysics. Also unforgettable is his habit of concluding the regular meetings of the Physiological Institute in Zurich with the reading of a poem. Along these lines, he admired, loved, and revered Shakespeare.
